# Development and validation of a risk stratification model for screening suspected cases of COVID-19 in China

**DOI:** 10.18632/aging.103694

**Published:** 2020-07-29

**Authors:** Jing Ma, Xiaowei Shi, Weiming Xu, Feifei Lv, Jian Wu, Qiaoling Pan, Jinfeng Yang, Jiong Yu, Hongcui Cao, Lanjuan Li

**Affiliations:** 1 Department of Laboratory Medicine, The First Affiliated Hospital, College of Medicine, Zhejiang University, Hangzhou 310003, China; 2 State Key Laboratory for The Diagnosis and Treatment of Infectious Diseases, National Clinical Research Center for Infectious Diseases, The First Affiliated Hospital, College of Medicine, Zhejiang University, Hangzhou 310003, China; 3 Collaborative Innovation Center for Diagnosis and Treatment of Infectious Diseases, Hangzhou 310003, China; 4 Taizhou Enze Medical Center (Group) Enze Hospital, Taizhou 318050, China

**Keywords:** severe acute respiratory syndrome coronavirus 2, COVID-19, risk stratification, model, suspected cases

## Abstract

How to quickly identify high-risk populations is critical to epidemic control. We developed and validated a risk prediction model for screening SARS-CoV-2 infection in suspected cases with an epidemiological history. A total of 1019 patients, ≥13 years of age, who had an epidemiological history were enrolled from fever clinics between January 2020 and February 2020. Among 103 (10.11%) cases of COVID-19 were confirmed. Multivariable analysis summarized four features associated with increased risk of SARS-CoV-2 infection, summarized in the mnemonic COVID-19-REAL: radiological evidence of pneumonia (1 point), eosinophils < 0.005 × 10^9^/L (1 point), age ≥ 32 years (2 points), and leukocytes < 6.05 × 10^9^ /L (1 point). The area under the ROC curve for the training group was 0.863 (95% CI, 0.813 - 0.912). A cut-off value of less than 3 points for COVID-19-REAL was assigned to define the low-risk population. Only 10 (2.70%) of 371 patients were proved to be SARS-CoV-2 positive, with a negative predictive value of 0.973. External validation was similar. This study provides a simple, practical, and robust screening model, COVID-19-REAL, able to identify populations at high risk for SARS-CoV-2 infection.

## INTRODUCTION

At the end of December 2019, an outbreak of pneumonia caused by a novel coronavirus (severe acute respiratory syndrome coronavirus 2, SARS-CoV-2) was reported in Wuhan, China [1]. Transmission takes place through respiratory droplets and other routes such as ocular surfaces [2–4]. This highly contagious virus spread rapidly to other cities of China, and gave rise to a global outbreak. As of Mar 23, 2020, over 300,000 cases of COVID-19 have been confirmed worldwide, and more than 10,000 have died. The number of confirmed cases is still increasing. One study estimates the basic reproductive number (R0) to be 2.68, and the epidemic doubling time to be 6.4 days [5]. The control of COVID-19 must include detection and isolation of latent infection. A considerable proportion of COVID-19 cases are infected by those who only had mild symptoms [6, 7]. COVID-19 patients have the highest viral load near symptom presentation [8]. Moreover, the rapid spread of COVID-19 has meant that large numbers of patients with suspicious symptoms are often crowded into fever clinics for diagnosis.

At present, cases are confirmed by a positive result with high-throughput sequencing or real-time reverse-transcriptase polymerase-chain-reaction (RT-PCR) assay of samples from nasal or pharyngeal swabs [9]. However, nucleic acid tests are not available to all suspected patients in pandemic areas due to the shortage of equipment and reagents [10, 11]. Testing for all cases with mild symptoms and/or an epidemiological history can lead to competition for resources. In addition, undiagnosed mild-type COVID-19 patients who were not properly isolated could become sources of infection as their viral load peaks near symptom presentation, which could explain the rapid spread of this epidemic [12]. A large proportion of infected cases continue to test negative for viral RNA, even after they develop clinical manifestations, and positive chest CT (computed tomography) results [13, 14]. This dilemma demands a fast and accurate model for early screening for SARS-CoV-2 infections to prioritize high-risk patients for clinical care, isolation, and contact tracking. Previous studies reported that a number of COVID-19 patients exhibit lymphopenia and thrombocytopenia [15–17]. Blood counts and high-sensitivity C-reactive protein (hsCRP) are commonly used for early identification of fever [18], and CT is used to assess pneumonia. These tests are simple and fast, and nearly all patients with fever or respiratory symptoms can be tested. We first compared alterations of hematological parameters between cases with and without SARS-CoV-2 infection, then developed and validated a novel score-based prognostic model (COVID-19-REAL) for SARS-CoV-2 infection.

## RESULTS

### Patient characteristics

**Figure 1:**
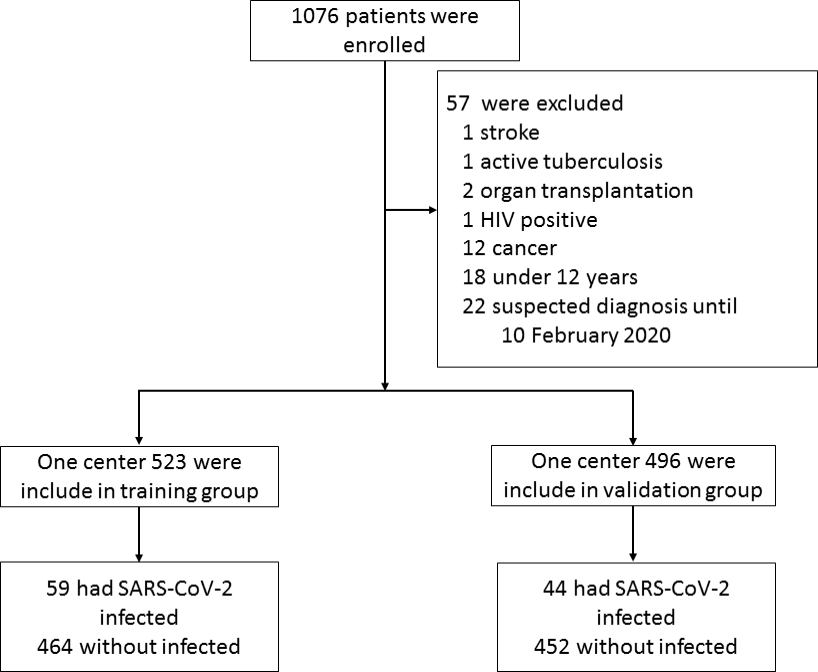
**Flowchart of patient selection.**

A total of 1019 patients were enrolled in this study out of the 1076 patients who presented to fever clinics until 5 February 2020. Fifty-seven patients were excluded, including one with stroke, two with organ transplantation, one with HIV, 12 with cancer, one with active tuberculosis, 18 with age < 12 years, and 22 unconfirmed cases until 10 February 2020 (Figure 1). Of the 1019 patients, 485 (48%) were female, and the median age was 34 years (range 13 to 91 years). The characteristics of the patients are shown in Table 1. All received sequencing or nucleic acid testing using RT- PCR; 103 (10.11%) tested positive for SAR-CoV-2 (Supplementary Table 1).

**Table 1:** Characteristics of patients in this study.

**Characteristic**	**Development group**	**Validation group**	**P-value**
Number	523	496	
Female	253 (48.38%)	232 (46.77%)	0.609
Age (years)	33 (24-45)	32 (26-40)	0.895
Symptom			
Fever	412 (78.78%)	367 (73.99%)	0.072
Dry cough	209 (39.96%)	171 (34.48%)	0.070
Fatigue	45 (8.60%)	43 (8.669%)	0.970
Pharyngalgia	84 (16.06%)	89 (17.94%)	0.424
Diarrhea	12 (2.29%)	13 (2.62%)	0.736
**Coexisting comorbidity**			
Hypertension	29 (5.54%)	34 (6.85%)	0.386
Cardiovascular diseases	6 (1.15%)	5 (1.01%)	0.83
Diabetes	11 (2.10%)	7 (1.41%)	0.48
Chronic lung disease	0 (0.00%)	3 (0.60%)	0.115
Chronic liver disease	11 (2.10%)	19 (3.83%)	0.103
Chronic renal disease	1 (0.19%)	2 (0.40%)	0.615
**Blood parameters**			
Leucocyte (109/L)	6.9 (5.30-8.80)	7.0 (5.20-9.03)	0.74
hsCRP (mg/L)	5.07 (0.90-15.95)	9.10 (2.75-22.56)	<0.001
Monocyte (109/L)	0.50 (0.40-0.70)	0.55 (0.41-0.76)	0.477
RBC (1012/L)	4.78 (4.44-5.22)	4.74 (4.37-5.14)	0.031
Hematocrit (%)	0.42 (0.40-0.46)	0.42 (0.39-0.46)	0.538
Lymphocyte (109/L)	1.30 (0.90-1.80)	1.25 (0.86-1.69)	0.592
MCH (pg)	30.30 (29.30-31.00)	30.30 (29.48-31.20)	0.074
MCHC (g/L)	339.00 (333.00-345.00)	339.00 (332.00-345.00)	0.251
MPV	10.00 (9.60-10.60)	10.00 (9.40-10.60)	0.04
Basophilic granulocyte (109/L)	0.02 (0.01-0.02)	0.02 (0.01-0.03)	<0.001
Eosinophil (109/L)	0.04 (0.01-0.08)	0.03 (0.01-0.09)	0.612
Hemoglobin (g/L)	143 (133-157)	144.00 (132-156)	0.318
PDW (%)	11.70 (10.80-12.85)	11.20 (10.10-12.60)	0.003
Platelet (109/L)	216 (181-256)	212 (173-256)	0.874
Platelet hematocrit (%)	0.22 (0.18-0.25)	0.21 (0.18-0.25)	0.37
Neutrophil (109/L)	4.70 (3.40-6.60)	4.75 (3.30-7.10)	0.7
Radiological evidence of pneumonia	92 (17.59%)	63 (12.70%)	0.03
Confirmed with COVID-19	59 (11.28%)	44 (8.87%)	0.202

Abbreviations: SARS-CoV-2: severe acute respiratory syndrome coronavirus 2; HsCRP: high-sensitivity C-reactive proteins; RBC: red blood cell; MCH: mean corpuscular hemoglobin; MPV: mean platelet volume; MCHC: mean corpuscular hemoglobin concentration; PDW: platelet distribution width; CT: chest computed tomography scan.

### Association factors for SARS-CoV-2 infection

**Figure 2:**
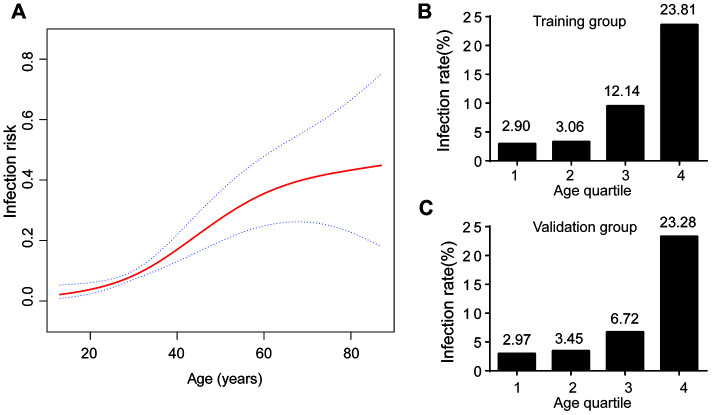
**Age and COVID-19 infection.** (**A**) The infection risk increased with increasing age; (**B**) Infection rate at age quartile in training group; (**C**) Infection rate at age quartile in validation group.

The association between age and infection rate is presented in Figure 2A. The rate of SARS-CoV-2 infection increased with age. After stratifying patients by age quartile, the positive rate of SARS-CoV-2 infection from first to fourth quartile was 2.90%, 3.06%, 12.14%, and 23.81% in the training group, and 2.97%, 3.45%, 6.72%, and 23.28% in the validation group (Figure 2B, C). The risk of infection in last two quartiles was relatively higher than the first two quartiles. The infection rate was lower (less than 5%) for patients with age < 32 years. Subgroup analyses were performed for patients with age ≥ 32 years to stratify those as high-risk population.

The factors associated with a positive result of SARS-CoV-2 infection in univariate analysis are shown in Table 2. Compared to non-COVID-19 patients, COVID-19 patients had a lower count of leukocytes (5.10×10^9^/L vs 7.15×10^9^/L, p < 0.001), monocytes (0.40×10^9^/L vs 0.55×10^9^/L, p < 0.001), lymphocytes (1.10×10^9^/L vs 1.30×10^9^/L, p = 0.02), eosinophils (0.01×10^9^/L vs 0.04×10^9^/L, p < 0.001), neutrophils (3.40×10^9^/L vs 5.00×10^9^/L, p < 0.001), and platelets (192×10^9^/L vs 220×10^9^/L, p < 0.001). They had a higher age (47 years vs 32 years, p < 0.001) in the training group, and similar characteristics were found in validation group (Supplementary Tables 2). After multivariate analysis, age, leukocytes, and eosinophils remained as significant factors; lymphocytes, leukocytes, monocytes, platelets, and neutrophils were not significant indicators (Table 2).

**Table 2:** Univariate and multivariate analyses of indicators for SARS-CoV-2 infection in training group.

**Variable**	**non-COVID-19 N = 464**	**COVID-19 N = 59**	**Univariate**		**Multivariate**	
			**OR (95% CI)**	**P-value**	**OR (95% CI)**	**P-value**
Age (years)	32 (23-42)	47 (38-56)	1.05 (1.04- 1.07)	<0.001	1.06 (1.04- 1.08)	<0.001
Leucocyte (109/L)	7.15 (5.70-9.03)	5.10 (4.05-6.05)	0.72 (0.63- 0.83)	<0.001	0.74 (0.64- 0.85)	<0.001
Monocyte (109/L)	0.55 (0.40-0.70)	0.40 (0.30-0.50)	0.06 (0.01- 0.24)	<0.001		
RBC (1012/L)	4.80 (4.45-5.24)	4.70 (4.25-5.01)	0.46 (0.27- 0.78)	0.004		
Lymphocyte (109/L)	1.30 (0.90-1.90)	1.10 (0.85-1.50)	0.57 (0.35- 0.91)	0.019		
Basophilic granulocyte (109/L)	0.02 (0.01-0.03)	0.01 (0.01-0.02)	0.00 (0.00- 45.46)	0.098		
Eosinophil (107/L)	4.00 (1.00-9.00)	1.00 (0.00-3.00)	0.88 (0.82- 0.95)	0.001	0.91 (0.85- 0.98)	0.009
Platelet (109/L)	220.00 (184.00-259.00)	192.00 (144.50-234.00)	0.99 (0.99- 1.00)	<0.001		
Neutrophil (109/L)	5.00 (3.60-6.80)	3.40 (0.80-22.20)	0.75 (0.65- 0.87)	<0.001		
Radiological evidence of pneumonia	68 (14.66%)	24 (40.68%)	3.99 (2.24- 7.13)	<0.001	4.00 (2.04- 7.86)	<0.001

Abbreviations: SARS-CoV-2: severe acute respiratory syndrome coronavirus 2; RBC: red blood cell; HsCRP: high-sensitivity C-reactive proteins; CT: chest computed tomography scan; CI: confidence interval; OR: odds ratio.

### A COVID-19 prediction model based on age, leukocyte, and eosinophil and radiological evidence of pneumonia

The AUROC value for the prediction of leukocytes and eosinophils in the training group for COVID-19 diagnosis were 0.747 and 0.729, respectively. This was comparable to the validation group, where the AUROC value for leukocytes and eosinophils were 0.763 and 0.772 (Supplementary Figure 1). Using Youden’s index, the optimal cut-off value for leukocytes and eosinophils were 6.05 × 10^9^/L and 0.005 × 10^9^/L.

**Figure 3:**
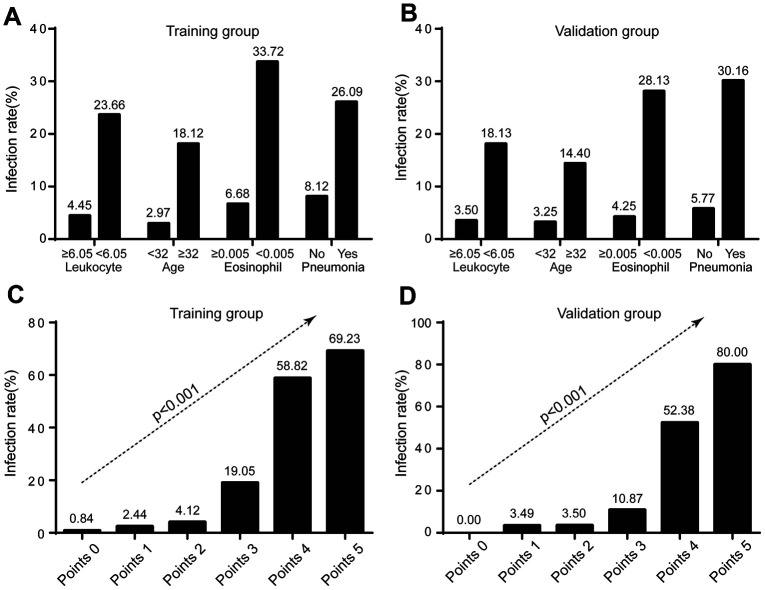
**Infection rate in risk stratification.** (**A**) Infection rate stratified by leukocyte, age, eosinophil, and radiological evidence of pneumonia in training group; (**B**) Infection rate stratified by leukocyte, age, eosinophil, and radiological evidence of pneumonia in validation group; (**C**) Infection rate according to COVID-19-REAL score in training group; (**D**) Infection rate according to COVID-19-REAL score in validation group.

Significantly higher infection rate was observed in those with leukocytes < 6.05×10^9^/L (23.66% vs 4.45% in leukocytes ≥ 6.05×10^9^/L), and eosinophils < 0.005×10^9^/L (33.72% vs 6.68% in eosinophils ≥ 0.005×10^9^/L) in the training group. The trend was consistent in the validation group, where the infection rate was 18.13% vs 3.5% for leukocyte subgroups, and 28.13% vs 4.25% for eosinophil subgroups (Figure 3).

Based on multivariate logistic regression analysis, the major criterion was age ≥ 32 years (2 point). Minor criteria included leukocytes < 6.05×10^9^/L (1 point), eosinophils < 0.005×10^9^/L (1 point), and radiological evidence of pneumonia (1 point) (Table 3). The model showed good discrimination (AUROC = 0.863, 95% CI, 0.81 - 0.91) and calibration. Internal verification shows AUROC = 0.863 (95% CI, 0.81 - 0.91) and external verification showed good discrimination (AUROC = 0.871, 95% CI, 0.82-0.93) (Table 4, Supplementary Figure 2)

**Table 3:** Multivariate analyses of indicators for SARS-CoV-2 infection in training group.

**Variable**	**OR (95% CI)**	**β Coefficient (95% CI)**	**P-value**	**Point score**
**Age (years)**				
<32 (n = 236)	1	1		
≥32(n = 287)	8.63 (3.60 - 20.64)	2.16 (1.28- 3.03)	<0.001	2
**Eosinophil (109/L)**				
>0.005 (n = 437)	1	1		
≤0.005 (n = 86)	4.92 (2.50 - 9.69)	1.59 (0.94 - 2.27)	<0.001	1
**Leucocyte (109/L)**				
>6.05 (n =337)	1	1		
≤6.05 (n =186)	6.23 (3.14 - 12.35)	1.83 (1.14 - 2.51)	<0.001	1
**Radiological evidence**				
No Pneumonia(n = 431)	1	1		
Pneumonia(n = 92)	3.73 (1.83 - 7.62)	1.32 (0.60 – 2.03)	<0.001	1

Abbreviations: SARS-CoV-2: severe acute respiratory syndrome coronavirus 2; CT: chest computed tomography scan; CI: confidence interval; OR: odds ratio.

**Figure 4:**
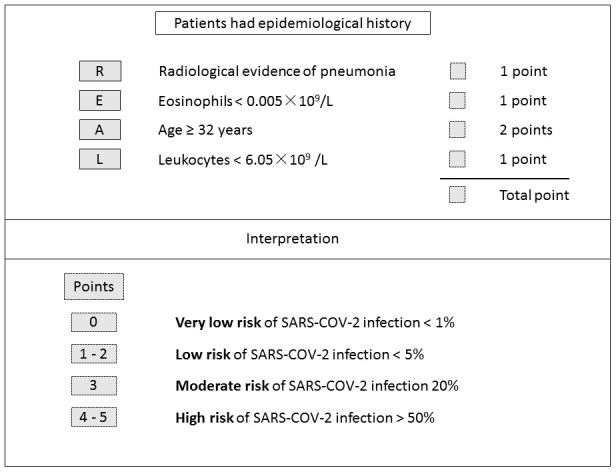
**COVID-19-REAL model for risk stratification of SARS-CoV-2 infection.**

The following four risk groups were developed: very low risk (0 point), with a risk of infection of 0.84%; low risk (1 - 2 points), with a risk of 3.57%; moderate risk (3 points), with a risk of 19.05%; and high risk (4 - 5 points), with a risk of 61.70%. For the validation group, the infection risk was 0% (0 point); 3.49% (1 - 2 points); 10.87% (3 points); and 55.32% (4 - 5 points) (Figure 4). A cut-off value of less than 3 points for COVID-19-REAL was used to stratify 371 out of 523 (70.94%) cases as low risk, of whom only 10 (2.70%) were infected with SARS-CoV-2 in the training group. The remaining 152 patients were classified as higher risk of infection; about 49 (32.24%) were infected with SARS-CoV-2. According to the cut-off value of 3 points, the sensitivity, specificity, positive predictive value, and negative predictive value was 0.778, 0.831, 0.322, and 0.973 respectively (Table 4).

**Table 4:** Performances of the risk stratification algorithm in the diagnosis of SARS-CoV-2 infection in training and validation groups.

**Group**	**AUROC (95% CI)**	**Specificity**	**Sensitivity**	**Positive PV**	**Negative PV**
Training group	0.863 (0.813-0.912)	0.778	0.831	0.322	0.973
Validation group	0.871 (0.816-0.925)	0.772	0.818	0.259	0.978

Abbreviations: SARS-CoV-2: severe acute respiratory syndrome coronavirus 2; CI: confidence interval; Positive PV: positive predictive value; Negative PV: negative predictive value.

## DISCUSSION

Beginning in mid-January 2020, a large number of people living in Wuhan left the area via public transportation due to Chinese New Year, leading to a dramatic increase in confirmed or suspected cases nationwide. The management of these suspected cases is of major concern. Nucleic acid testing is currently the main diagnostic method, but the sensitivity and specificity of nucleic acid tests are yet to be verified, and the overall detection rate is constrained by virus concentration and sampling method. Another problem is that some patients with positive chest CT images test negative for COVID-19 by RT-PCR [14]. With such issues in mind, we proposed a robust, high-throughput screening model to help prioritize high-risk patients. We used the data of routine blood tests and CT images to develop a score system (COVID-19-REAL) that can stratify patients into risk groups. Suspected cases with 0 - < 3 points had a predicted probability of 99.16% in training and 97.3% in validation groups for not being infected by SARS-CoV-2. This risk classification can be employed by clinicians and medical institutions, especially those with inadequate detection reagents or equipment, to make rational allocation of resources.

Previous investigations have revealed valuable information about demographics for COVID-19. Most patients with COVID-19 are older [16]. We first stratified patients according to age. Two earlier studies stated the median age of the patients was 56 and 59 years [15, 19]. In our study, the median age was 47 years. We found the risk of infection significantly increased with age, from less than 3% to over 23% from the first to last quartile.

The level of leukocytes, monocytes, lymphocytes, eosinophils, neutrophils, and platelets was dramatically lower in COVID-19 patients. Our results are consistent with previous research that patients exhibited leukopenia, lymphopenia, and thrombocytopenia after SARS-CoV-2 infection [15, 20]. Some researchers suggested a decreased level of white blood cells could serve as an auxiliary diagnosis [20]. Similar patterns emerged in SARS-CoV, with cases of lymphopenia and neutropenia [21, 22], and decreased levels of leukocytes and platelets [23]. A SARS-CoV model showed that neutrophils, lymphocytes, and leukocytes were significantly reduced the day after infection [24]. In a SARS-CoV MA15 infection model, the decrease of peripheral blood cells was explained by inflammatory cell infiltration to the lungs [25]. The N protein of SARS-CoV enhances eosinophilic infiltration into the lungs and aggravates lung inflammation [26]. Lung lesions were the most important feature of SARS-CoV-2 infections [20], and eosinophilopenia may indicate a poor prognosis of COVID-19 [27]. These results shed light on the neglected role that eosinophils might play in the progression of respiratory disease.

To better stratify SARS-CoV-2 infection risk for the suspected cases, four criteria including leukocytes < 6.05×10^9^ /L (1 point), eosinophils < 0.005×10^9^/L (1 point), radiological evidence of pneumonia (1 point), and age ≥ 32 years (2 point) were used to determine the likelihood of SARS-CoV-2 infection. We defined four risk groups: very low risk (0 point), low risk (1 - 2 points), moderate risk (3 points), and high risk (4 - 5 points). According to the cut-off value that was assigned as less than 3 points of COVID-19-REAL score, the number of suspected cases who required priority examination and hospitalization decreased by 70.94% and 71.98%, while maintaining a false negative rate of 2.70% and 2.24% in training and validation group, respectively.

Clinical decision models have been explored to predict infection of SARS-CoV-2. Sun et al. [28] studied 788 cases in Singapore to identify populations at high risk for COVID-19. From their large population-based study, a model that combined laboratory blood tests, clinical findings, and radiology was proposed, and the AUROC was 0.88 (95% CI: 0.83- 0.93). Similar to our cohort, those authors found that eosinophils and CT imaged pneumonia were strong predictors. However, their conclusions were limited by a lack of external verification, clinical inapplicability caused by redundant parameters, and missing data in laboratory blood tests.

The advantage of present study is that a simple and applicable prediction model, COVID-19-REAL, which combines age, radiological image, and two functionally related hematological indicators (i.e., leukocytes and eosinophils) has been developed to stratify and distinguish between high- and low-risk populations suspected of SARS-CoV-2 infection. This evaluation of suspected cases based on age, radiological image, and two dichotomous criteria could be easily implemented in routine clinical practice. In clinical settings where resources and testing kits are limited, patients with advanced respiratory symptoms are usually tested first. However, those undiagnosed mild-type COVID-19 patients who were not properly isolated would become sources of infection as the viral load peaked near symptom presentation. This score system will be of great help for early infection screening and offer more information for physicians to help prioritize high-risk patients.

There are limitations in current study. Our training and validation data comes from China; their applicability to Western populations must be separately evaluated. The results were obtained from people over 12 years of age, and may not be applicable to younger people. Only routine tests including hsCRP, radiological image, and blood cell count were performed, and other hematological indicators including liver and kidney function are lacking.

In conclusion, this study provides a simple, practical, and robust screening model (COVID-19-REAL) to identify high risk populations for SARS-CoV-2 infection. This prediction model will help reduce the burden on hospitals in pandemic areas and help them allocate resources more rationally.

## MATERIALS AND METHODS

### Patients

Suspect cases of COVID-19 with age ≥13 years with an epidemiological history were included from fever clinics of the First Affiliated Hospital, College of Medicine, Zhejiang University and Taizhou Enze Medical Center (Group), Enze Hospital, between 23 January 2020 and 5 February 2020. All suspected cases received sequencing or RT-PCR assay for SARS-CoV-2. According to National Health Commission, an epidemiological history of COVID-19 is defined as follows: within 14 days before the onset of the disease (1) there were tourism or residence histories of Wuhan or its surrounding areas, or other communities with confirmed cases; (2) there were contacts with confirmed cases of COVID-19; (3) there were contacts with suspected cases (having fever or respiratory symptoms) from Wuhan or its surrounding areas, or other communities with confirmed cases; (4) one confirmed case was found in an enclosed environment (such as a family house, a construction site, an office, etc.), with one or more cases of fever/respiratory tract infection re found at the same time

The patient-selection process is shown in Figure 1. The COVID-19 cases were all confirmed by sequencing or RT-PCR assay [9]. The RT-PCR was mainly performed using a commercial kit for SARS-CoV-2 detection (BoJie, Shanghai, China) which was approved by China Food and Drug Administration. We excluded patients with HIV infection, cancer, organ transplantation, stoke, active tuberculosis, severe and critical COVID-19 patients according to the National Health Commission [17], and suspected cases without confirmed laboratory evidence until 10 February 2020. The study was approved by the Ethics Committee of the First Affiliated Hospital, College of Medicine, Zhejiang University, and complied with the ethical guidelines of the Declaration of Helsinki. The researchers only analyzed anonymous data, so informed consent was waived. Age, gender, laboratory assessments consisting of hsCRP, complete blood count, and radiological images were obtained from electronic medical records. Radiological evidence of pneumonia was defined as lung consolidation and/or ground-glass opacity [20]. The images were reviewed independently by two radiologists, and if there were disagreements, a third radiologist would perform further examination.

### Statistical analysis

Continuous variables were expressed as medians and interquartile range (IQR), and were compared by t-test or Mann–Whitney U-test. Chi-squared test or Fisher’s exact test was used to compare categorical variables and expressed as percentages. Generalized linear models with a logit link were used to test the association between age and the risk of COVID-19 infection. Univariate and multivariate analyses were performed to identify indicators of COVID-19 patients. Variables with *P <* 0.1 in a univariate analysis were then included in a forward stepwise regression model. A score for the final model was developed by rounding the coefficients of the logit model. Predicted and observed risk was calculated for each score. The area under receiver operating characteristic (AUROC) curve was used to assess the accuracy of different scores in diagnosis power. Internal validation was performed using a bootstrap procedure with 500 bootstrapped samples. The Youden’s index was used to determine the optimal cut-off level for predicting clinical outcomes. All statistical analysis was performed by Statistical Package for the Social Sciences version 19.0 (International Business Machines Corporation, Armonk, NY) and R version 3.4 (R Foundation, Vienna, Austria). All tests were two tailed and P < 0.05 was considered to indicate statistical significance.

**ACKNOWLEDGMENTS**

This work was supported by the National Key Research and Development Program of China (No. 2016YFA0101001).

## Supplementary Material

Supplementary Figures

Supplementary Tables
