# Supplementary material for: Development and validation of a risk stratification model for screening suspected cases of COVID-19 in China
Source: Aging (Albany NY). 2020 Jul 29;12(14):13882–94. doi: 10.18632/aging.103694 (PMC7425460; doi:10.18632/aging.103694)
Supplement: Supplementary Figures [file aging-12-103694-s002..pdf]

SUPPLEMENTARY FIGURES

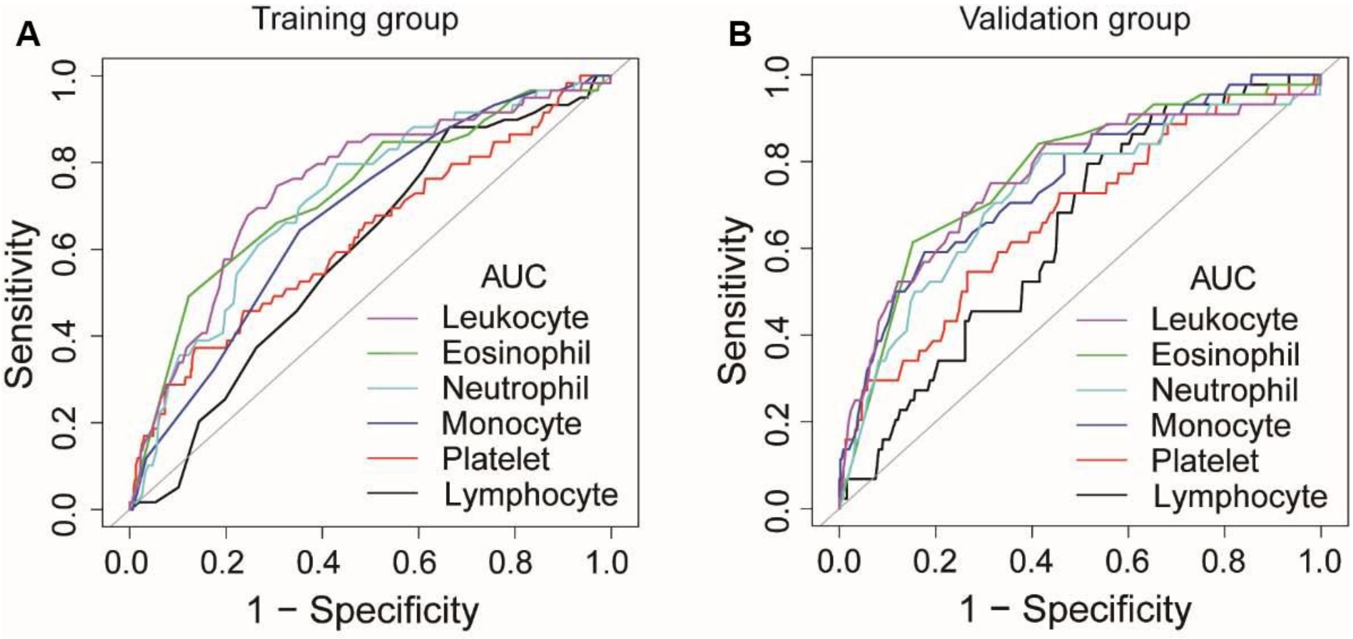

Supplementary Figure 1. AUROC of leukocyte, monocyte, lymphocyte, eosinophil, neutrophil and platelets in COVID-19 diagnosis. (**A**) training group; (**B**) validation group.

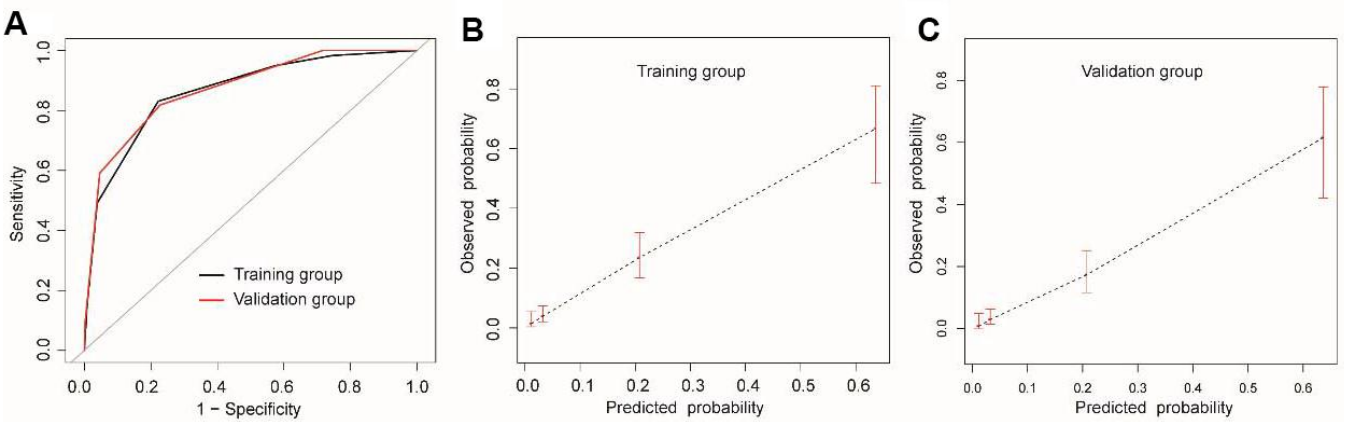

Supplementary Figure 2. AUROC of model in COVID-19 diagnosis (**A**), and Calibration chart for predicted versus observed probability (**B**, **C**) in training and validation group.
