# Supplementary material for: Development and validation of a risk stratification model for screening suspected cases of COVID-19 in China
Source: Aging (Albany NY). 2020 Jul 29;12(14):13882–94. doi: 10.18632/aging.103694 (PMC7425460; doi:10.18632/aging.103694)
Supplement: Supplementary Tables [file aging-12-103694-s001..pdf]

SUPPLEMENTARY TABLES

Supplementary Table 1. Characteristics of patients with SARS-CoV-2 infected.

Characteristic	Development group	Validation group	P-value
Number	59	44	
Female	24 (40.68%)	15 (34.09%)	0.495
Age (years)	47 (38-56)	48 (35-57)	0.812
Symptom			
Fever	37 (62.71%)	32 (72.73%)	0.285
Dry cough	24 (40.68%)	18 (40.91%)	0.981
Fatigue	6 (10.17%)	4 (9.09%)	0.855
Pharyngalgia	14 (23.73%)	8 (18.18%)	0.497
Blood parameters			
Leucocyte (109/L)	5.10 (4.05-6.05)	4.50 (3.63-6.03)	0.738
hsCRP (mg/L)	10.17 (2.62-21.88)	14.80 (5.35-30.10)	0.043
Monocyte (109/L)	0.40 (0.30-0.50)	0.35 (0.27-0.52)	0.224
RBC (1012/L)	4.70 (4.25-5.01)	4.69 (4.22-5.01)	0.88
Hematocrit (%)	0.42 (0.38-0.45)	0.42 (0.38-0.44)	0.668
Lymphocyte (109/L)	1.10 (0.85-1.50)	1.10 (0.73-1.30)	0.134
MCH (pg)	30.60 (29.65-31.30)	30.15 (29.30-31.33)	0.439
MCHC (g/L)	341.00 (334.00-346.50)	341.00 (334.50-348.00)	0.939
MPV	10.40 (9.90-10.85)	10.35 (9.95-11.00)	0.707
Basophilicgranulocyte (109/L)	0.01 (0.01-0.02)	0.01 (0.00-0.01)	0.066
Eosinophil (109/L)	0.01 (0.00-0.03)	0.00 (0.00-0.02)	0.757
Hemoglobin (g/L)	144.00 (129.00-153.00)	143.00 (129.00-152.00)	0.641
PDW (%)	12.00 (11.20-13.10)	11.90 (10.73-13.03)	0.403
Platelet (109/L)	192.00 (144.50-234.00)	177.00 (140.00-226.00)	0.4
Platelet hematocrit (%)	0.20 (0.15-0.23)	0.18 (0.15-0.24)	0.453
Neutrophil (109/L)	3.40 (2.60-4.45)	3.00 (2.18-4.15)	0.981
Radiological evidence of pneumonia	24 (40.68%)	19 (43.18%)	0.799

Abbreviations: HsCRP: high-sensitivity C-reactive proteins; RBC: Red Blood Cell; MCH: mean corpuscular hemoglobin; MPV: Mean platelet volume; MCHC: mean corpuscular hemoglobin concentration; PDW: Platelet distribution width; CT, chest computed tomography scan.

Supplementary Table 2. Characteristics of patients visited fever clinics.

Characteristic	**Non-COVID-19 infected**	**COVID-19 infected**	***P*-value**
Number	452	44	
Female	217 (48.01%)	15 (34.09%)	0.077
Age (years)	31 (25-38)	48 (35-57)	<0.001
Symptom			
Fever	335 (74.12%)	32 (72.73%)	0.841
Dry cough	153 (33.85%)	18 (40.91%)	0.347
Fatigue	39 (8.63%)	4 (9.09%)	0.917
Pharyngalgia	81 (17.92%)	8 (18.18%)	0.966
Blood parameters			
Leucocyte (10^9/L)	7.20 (5.50-9.50)	4.50 (3.63-6.03)	<0.001
hsCRP (mg/L)	8.80 (2.40-22.22)	14.80 (5.35-30.10)	0.547
Monocyte (10^9/L)	0.57 (0.43-0.78)	0.35 (0.27-0.52)	<0.001
RBC (10^{12}/L)	4.74 (4.38-5.14)	4.69 (4.22-5.01)	0.068
Hematocrit (%)	0.42 (0.40-0.46)	0.42 (0.38-0.44)	0.042
Lymphocyte (10^9/L)	1.29 (0.87-1.76)	1.10 (0.73-1.30)	0.004
MCH (pg)	30.30 (29.50-31.20)	30.15 (29.30-31.33)	0.442
MCHC (g/L)	339.00 (332.00-344.00)	341.00 (334.50-348.00)	0.046
MPV	9.95 (9.30-10.60)	10.35 (9.95-11.00)	<0.001
Basophilicgranulocyte (109/L)	0.02 (0.01-0.03)	0.01 (0.00-0.01)	<0.001
Eosinophil (109/L)	0.03 (0.01-0.09)	0.00 (0.00-0.02)	0.002
Hemoglobin (g/L)	144.00 (132.00-156.00)	143.00 (129.00-152.00)	0.177
PDW (%)	11.20 (10.10-12.45)	11.90 (10.73-13.03)	0.15
Platelet (109/L)	214.00 (176.00-260.00)	177.00 (139.75-226.00)	<0.001
Platelet hematocrit (%)	0.22 (0.18-0.25)	0.18 (0.15-0.24)	0.002
Neutrophil (109/L)	4.90 (3.50-7.22)	3.00 (2.18-4.15)	<0.001
Radiological evidence of pneumonia	44 (9.73%)	19 (43.18%)	<0.001

Abbreviations: HsCRP: high-sensitivity C-reactive proteins; RBC: Red Blood Cell; MCH: mean corpuscular hemoglobin; MPV: Mean platelet volume; MCHC: mean corpuscular hemoglobin concentration; PDW: Platelet distribution width; CT, chest computed tomography scan.
